# Response of HER2 positive gallbladder adenocarcinoma to trastuzumab emtansine (T-DM1): a case report and literature review

**DOI:** 10.3389/fonc.2026.1844516

**Published:** 2026-09-09

**Authors:** Wei Ling, Juxiang Zang, Fang Xiang, Xiufeng Liu, Shudong Chen

**Affiliations:** 1 Pharmacy Department, Jinling Hospital, Medical School of Nanjing University, Nanjing, China; 2 Oncology Department, Jinling Hospital, Medical School of Nanjing University, Nanjing, China

**Keywords:** ADC, biliary tract carcinoma, disease-free survival, HER2-positive, T-DM1

## Abstract

The incidence of biliary tract carcinoma (BTC) is gradually increasing, particularly in Asia where it is most common. Compared with other tumors, it has a low incidence but high mortality and is detected at an advanced stage. Metastatic BTCs have fewer options for systemic therapy and limited treatment options beyond symptomatic therapy for patients who have progressed on first-line therapy. There is therefore a need to explore a biomarker-guided personalized treatment approach. This report describes the case of a 46-year-old middle-aged man with human epidermal growth factor receptor 2 (HER2)-positive metastatic gallbladder adenocarcinoma who was treated preoperatively with two cycles of gemcitabine, oxaliplatin, lenvatinib, and sintilimab; the best objective response was stable disease. Subsequent next-generation sequencing showed positive HER2 gene mutation and the patient was treated with trastuzumab emtansine in combination with sintilimab. After 2 cycles, the lesion was reduced in size and surgical resection was achieved, and the patient underwent radical cholecystectomy for gallbladder cancer (GBC), with a disease-free survival of 13 months after surgery. This case demonstrates the efficacy of trastuzumab emtansine in the treatment of locally advanced GBC, suggesting that HER2 mutation is important in GBC and that anti-HER2 drug therapy may be an option for the systemic treatment of GBC.

## Introduction

Biliary tract carcinoma (BTC) is a type of tumor with a high degree of malignancy, characterized by a low incidence and a high mortality rate. BTC mainly includes gallbladder cancer (GBC), intrahepatic cholangiocarcinoma, and extrahepatic cholangiocarcinoma, accounting for approximately 3% of all digestive system tumors (1, 2). Recent studies have found that GBC shows a high-incidence trend among Asian populations (3). BTC is insidious, and patients are often ineligible for surgery when diagnosed (4). BTC exhibits prominent profibrotic and proliferative features, which compromise the diagnostic accuracy of cytological and pathological approaches. Therefore, genomic sequencing and molecular subtyping play an important role in its clinical management (5).

Currently, research into BTC driver genes such as human epidermal growth factor receptor 2 (HER2), FGFR2, PIK3CA, IDH1, BRAF, etc. has progressed rapidly in recent years. Drugs targeting the above driver genes have shown good efficacy in clinical trials (5, 6). With the widespread use of next-generation sequencing (NGS) technology, relevant studies have found that genotyping of BTC tends to differ in different parts of the body and that HER2 overexpression is present in about 10% of GBC patients (7). However, this prevalence exhibits significant geographic variation. For instance, Asian populations tend to demonstrate higher HER2-positive rates, which may be associated with both genetic and environmental factors (8). Several reports have described two distinct factors that may promote the development of HER2-positive gallbladder cancer. The first is the anatomical anomaly known as pancreaticobiliary maljunction; the second is long-term exposure to aflatoxins – a well-known environmental carcinogen (9, 10). The HER2 gene is a member of the epidermal growth factor receptor family, and both amplification and overexpression of HER2 can occur in many types of tumors (11). In GBC, HER2 positivity is associated with a more aggressive tumor phenotype, shorter overall survival, and higher recurrence rates. This biomarker serves not only as a prognostic indicator but also as a potential therapeutic target (8). In the systemic treatment of breast and gastric cancer, pharmacological treatment of HER2 mutations has become an important tool (6, 10). However, studies of HER2-directed therapy for BTC have been mostly restricted to small clinical trials in patient populations unselected for HER2 positivity (12).

In this report, we present a case of advanced HER2-positive GBC treated with trastuzumab emtansine (T-DM1) and evaluate the therapeutic efficacy of this agent in the patient. This case study holds significant clinical implications. It provides guiding insights into the application of T-DM1 in HER2-positive BTCs, underscores the potential of precision oncology in rare and refractory cancers, and offers valuable treatment references for similar cases with limited therapeutic options.

## Case report

**Figure 1:**
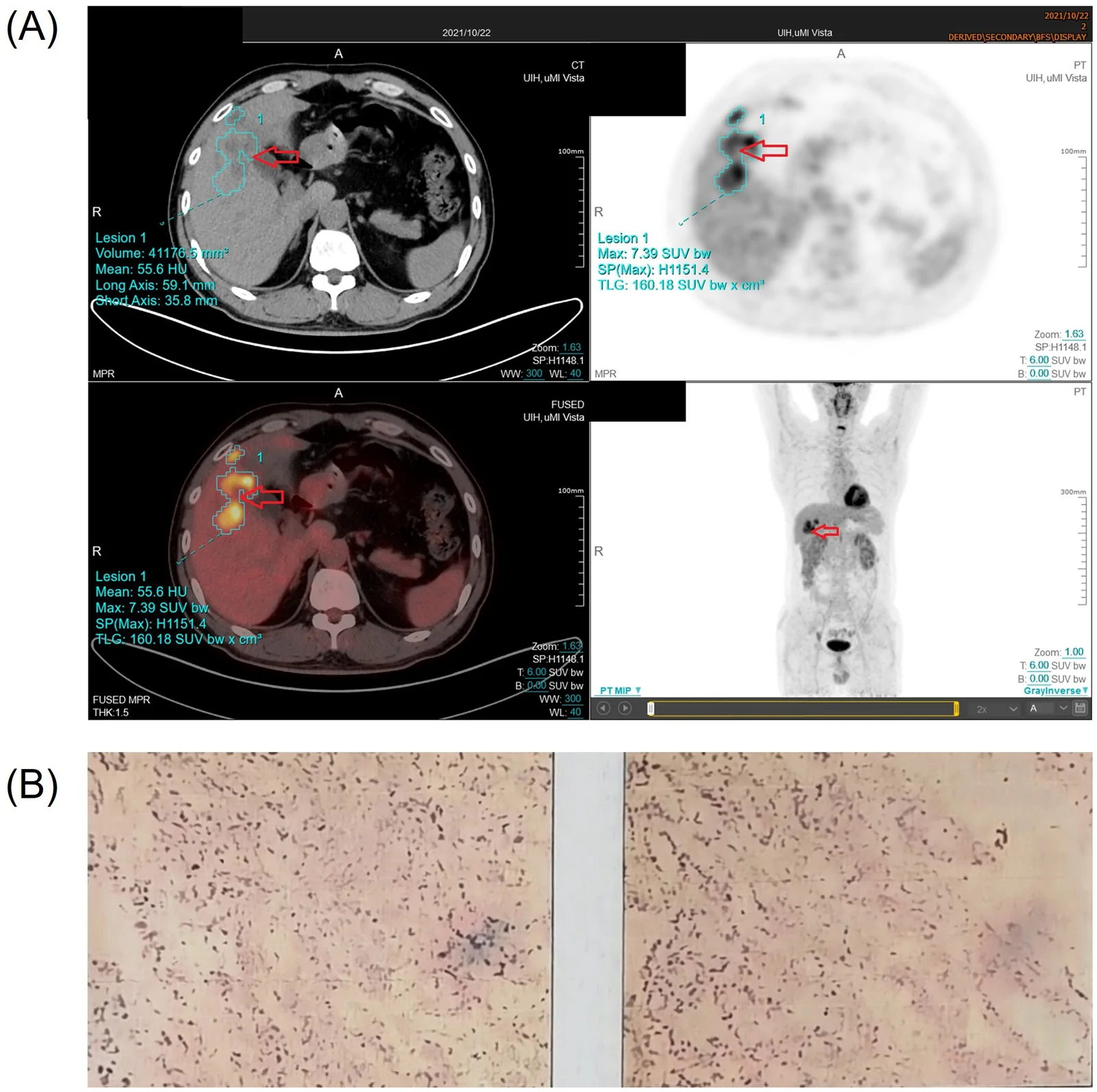
Patient admission examination status. **(A)** Local irregular thickening of the gallbladder wall, accompanied by multiple low-density foci in the adjacent liver parenchyma, suggesting gallbladder cancer with multiple metastases in the liver. **(B)** The results of immunohistochemical staining (for HER2 detection).

A 46-year-old male construction supervisor, with no history of tobacco use or alcohol consumption. He was referred for management of a gallbladder tumor. The case was discovered incidentally during an abdominal ultrasound examination in October 2021. Positron Emission Tomography-Computed Tomography (PET-CT) showed a localized irregular thickening of the gallbladder wall with multiple hypodense foci in the adjacent liver parenchyma, which was considered to be gallbladder carcinoma with multiple intrahepatic metastases (**Figure 1A**). Blood tests showed abnormalities in carcinoembryonic antigen (CEA) and carbohydrate antigen 125 (CA 125) (CEA = 207.1 ng/mL, CA 125 = 152.2 U/mL), while CA 19–9 and CA 50 were normal (CA19-9 = 10.0 U/mL, CA 50 = 3.0 U/mL). In October 2021, a puncture biopsy of the liver was performed in the medical oncology department of our hospital, with pathology suggestive of adenocarcinoma, and in conjunction with clinical considerations of bile duct or gallbladder origin. The immunohistochemical results of the puncture indicated HER2 (–) (**Figure 1B**). According to the UICC/AJCC (8th edition, 2017) diagnostic criteria for biliary tract malignancies, the patient was pathologically confirmed as having gallbladder adenocarcinoma. PET-CT revealed tumor invasion into adjacent hepatic tissue without definitive evidence of lymph node metastasis or distant metastasis. The patient was diagnosed with stage IVB (T4N0M1) GBC.

**Figure 2:**
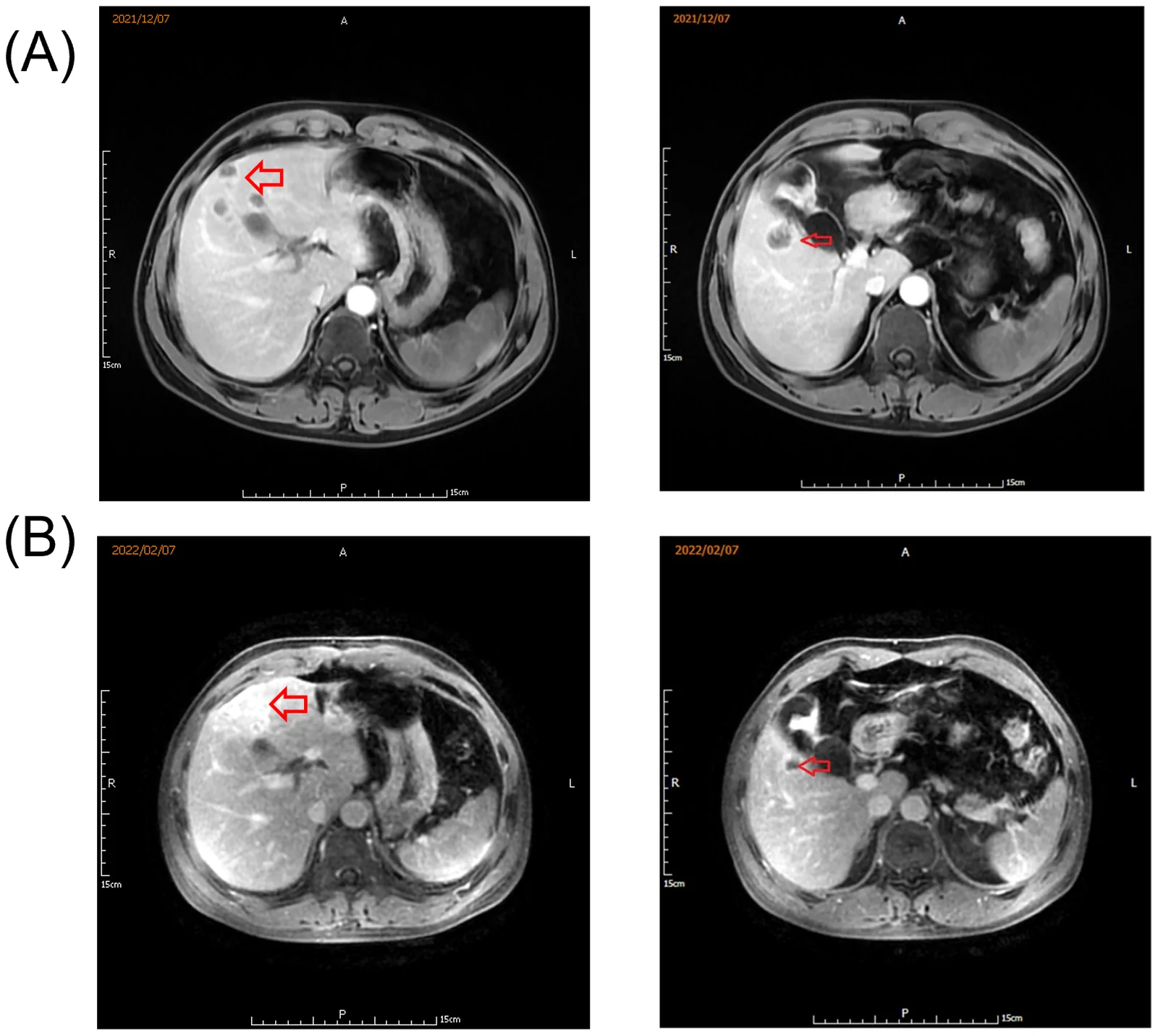
Serial abdominal MRI images of hepatic metastasis before and after T-DM1 plus sintilimab therapy. **(A)** Abdominal MRI obtained after two cycles of gemcitabine, oxaliplatin, lenvatinib and sintilimab therapy; no obvious reduction of the hepatic lesion was observed. **(B)** Abdominal MRI after two cycles of trastuzumab emtansine combined with sintilimab treatment. Obvious qualitative shrinkage of the target lesion can be appreciated. Precise quantitative assessment of tumour size was not feasible due to inconsistent slice positioning and respiratory phases across scans.

Multidisciplinary Treatment (MDT) suggested that the patient was at high risk of surgical recurrence and metastasis, and surgery was not recommended; it was suggested that two cycles of internal medicine therapy should be given first, and follow-up treatment should be discussed according to the changes in the lesions. He received two cycles of the GEMOX regimen (gemcitabine 1400 mg in 100 mL NS, day 1, day 8 and oxaliplatin 150 mg in 500 mL GS, day 1) combined with lenvatinib (8 mg qd) and sintilimab (200 mg in 100 mL NS, day 8) on 27 October 2021 and 19 November 2021. After two cycles, magnetic resonance imaging (MRI) scans showed no significant reduction in the size of the lesions (**Figure 2A**). Blood tests showed a decrease in tumor markers (CEA = 123 μg/L, CA 125 = 62.3 U/mL). The overall assessment of efficacy was stable disease. The patient had high expectations of treatment, so another MDT was held to discuss and recommend refinement of relevant genetic testing. Targeted gene-capture NGS (reference genome GRCh37/hg19) revealed tumor proportion score  < 1%, combined positive score (CPS)  = 1, microsatellite-stable (MSS), tumor mutational burden (TMB)  = 4.87 mutations/Mb, BRAF p.G466V, heterozygous HLA-I (A, B, C), and a somatic missense mutation in exon 8 of the ERBB2 gene. The variant was located at chr17:37868208 (reference transcript NM_004448.4), c.929C>T, p.S310F, with a variant allele frequency of 29.68%. No ERBB2 amplification or copy-number gain was detected.

**Figure 3:**
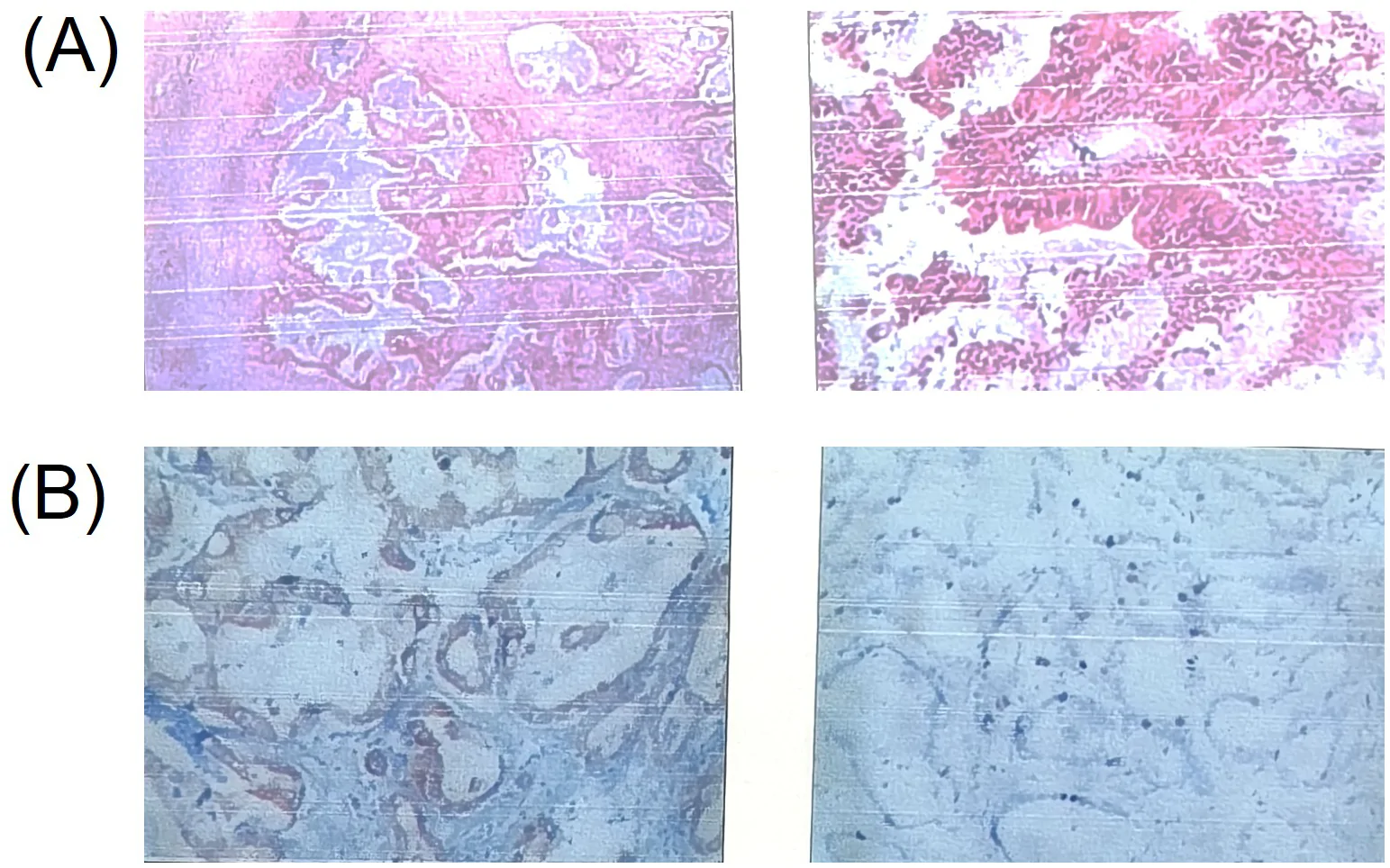
**(A)** Surgical pathology H&E staining. Gallbladder mucinous adenocarcinoma, moderately differentiated, with cancer tissue infiltrating the entire gallbladder wall and involving the liver parenchyma, visible nerve invasion, no intravascular cancer thrombus found, and no residual cancer tissue found in the gallbladder duct stump. No residual cancer tissue was found at the liver resection margin. Half of the 13a group lymph nodes showed cancer tissue metastasis, while 0/4 duodenal ligament lymph nodes showed no cancer tissue metastasis. **(B)** Immunohistochemical staining results: CK7 (+), CK19 (+), CEA (+), Ki-67 (20%+), CD34 (no tumor thrombus found in vessels).

Although this test is not covered by medical insurance in China, the NCCN Guidelines for the Diagnosis and Treatment of Biliary Tract Malignancies in the United States list NGS technology as a supplementary testing method. After repeated communication with the patient and his family, the patient insisted on trying T-DM1 therapy. Adjustment to T-DM1 (200 mg in 100 mL NS, day 1) in combination with sintilimab (200 mg in 100 mL NS, day 2) was initiated on 24 December 2021. After two cycles of treatment, the MRI (**Figure 2B**) was reviewed and showed that the lesion was smaller than before. Blood tests showed tumor markers continued to fall (CEA = 40.30 μg/L, CA 125 = 21.93 U/mL), while CA 19–9 was within normal range (CA 19-9 = 0.2 U/mL). Following MDT consultation, this patient was deemed to qualify for surgery. On 18 February 2022, he underwent radical surgery for GBC in our Oncological Surgery Department. Main surgical procedure: A Kocher incision was made to free the duodenum and remove No. 13a lymph nodes, and rapid pathological examination suggested tumor metastasis. First, liver and duodenal ligament lymph node dissection was performed to expose the common hepatic artery, gastroduodenal artery, left gastric artery, splenic veins, and common bile duct, and remove surrounding adipose and lymphatic tissue. The liver resection was performed again: the hepatic circular ligament was detached; the right lobe triangular ligament and inferior vena cava were sharply separated; the right liver was freed; the liver parenchyma was cut open along the right side of the hepatic circular ligament with an ultrasonic knife; the G46 branch was ligated and detached; the ischemic line of segment S4b was identified; the liver parenchyma was detached along the ischemic line; the main hepatic vein was ligated and detached; the liver section was expanded on the right side; the S5 branch was exposed, ligated and detached; the ischemic line of segment S5 was identified; the liver parenchyma was detached along the ischemic line; the S4b/S5 segment of the liver and gallbladder were completely removed; and the specimen was removed. Specimen incision examination: A lump at the bottom of the gallbladder, hard in texture, with a white cut surface. Scattered cancer nodules with a maximum diameter of about 2 cm were observed in the liver. During the surgery, the left and right halves of the liver were selectively blocked once each, with a total duration of about 30 minutes. The patient safely returned to the ward after the surgery. Pathological findings revealed mucinous adenocarcinoma, moderately differentiated, with cancerous tissue infiltrating the entire gallbladder wall, involving the intrahepatic parenchyma, with visible nerve invasion, 1/2 group 1A lymph nodes (+), and 0/4 lymph nodes of the duodenal ligament (+) (**Figure 3**).

**Figure 4:**
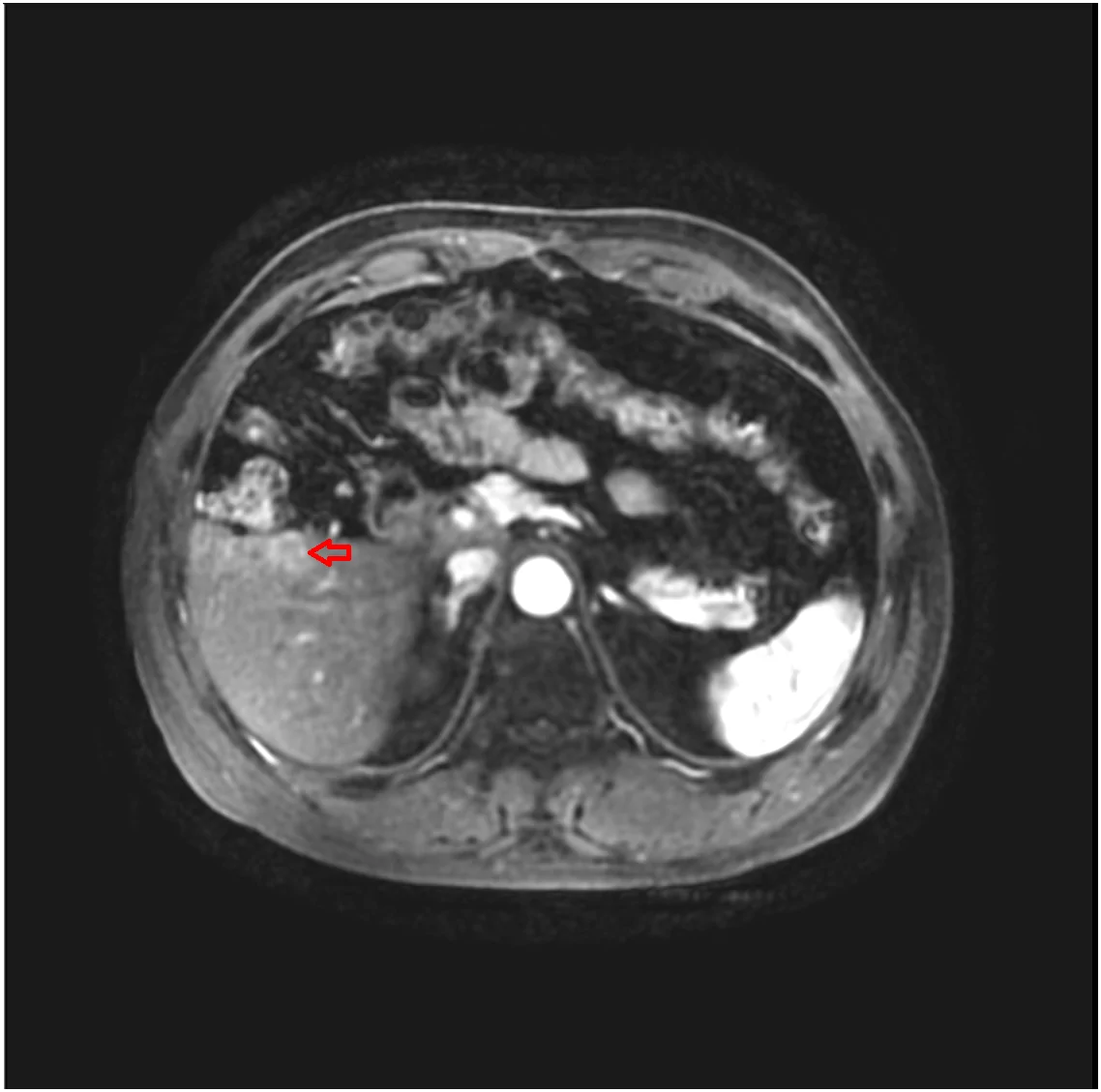
Postoperative MRI images of the patient, showing a new circularly enhanced lesion at the margin of the surgical area in the right lobe of the liver, suggestive of metastasis.

Postoperatively, 2 cycles of adjuvant chemotherapy with the GEMOX regimen (gemcitabine 1400 mg in 100 mL NS, day 1, day 8 and oxaliplatin 150 mg in 500 mL GS, day 1) were used. During chemotherapy, the patient developed a rash, itchy skin, nausea, vomiting, loss of appetite and malaise, which improved with symptomatic management, and the last chemotherapy was given on 19 April 2022. During the treatment period, the patient remained fully alert and communicative, with normal cognitive function. The patient reported fair appetite and sleep quality, experiencing mild postprandial abdominal distension but maintaining normal bowel and bladder function. After surgery, the patient had mild postprandial abdominal distension, could barely perform normal activities, and the Karnofsky Performance Status (KPS) score was 80. In February 2023, a blood test showed a slight increase in CEA (CEA = 10.505 ng/mL). On 9 March 2023, an upper abdominal MRI was performed (**Figure 4**), which showed a new circular enhancing focus at the surgical margin of the right lobe of the liver and a possible metastasis. The newly identified lesion demonstrated ring-like enhancement on imaging, which was confirmed by a radiologist. This radiographic feature strongly suggested metastatic disease. The patient was readmitted for continuation of treatment. Physical examination revealed a weight gain of 4 kg compared with five months previously. Diagnosis: post-resection of moderately differentiated adenocarcinoma of the gallbladder (intrahepatic metastasis), KPS 80. In conjunction with the history of previous medication, treatment with T-DM1 (200 mg in 250 mL NS, day 1) and sintilimab (200 mg in 250 mL NS, day 2) was continued. For the purposes of this report, written informed consent was obtained from the patient to publish the images and clinical details.

## Discussion

GBC is the most common form of BTC, accounting for 80-95% of BTC, and has a low survival rate (13). Currently, radical surgical resection after systemic therapy is the only hope for long-term survival for patients with inoperable biliary tract cancers. After conventional chemotherapy, fewer cases meet the criteria for surgery (14). Clinical trials of targeted therapy for GBC started late, although there have been some clinical trials evaluating the efficacy of targeted agents for GBC as monotherapy or combination therapy as a treatment option. However, no relevant targeted drugs have been approved for the treatment of advanced GBC in China (15). A previous study on the relationship between HER2 gene expression and clinical features in GBC found that patients with HER2 positivity were associated with poor prognostic factors, such as degree of tumor differentiation, neural invasion, lymph node metastasis, and confirmed that, in contrast to HER2-negative GBC patients, HER2-positive GBC patients had a higher frequency of distant metastases and a shorter median survival (16). This suggests a potential use of anti-HER2 agents for our patients with HER2-positive GBC. The patient’s HER2 immunohistochemistry (IHC) showed (–), while NGS results indicated a HER2 mutation. The potential reasons for the discrepancy in test results may include: the absence of a defined HER2-positive threshold for biliary tract malignancies. Currently, the criteria for HER2 positivity mainly refer to those for breast cancer and gastroesophageal junction tumors (17). There are diverse expression patterns of HER2 positivity in biliary tract malignancies, whereas IHC can only identify overexpression or amplification (18). In the NCCN guidelines summarizing HER2 genetic testing methods for biliary tract malignancies, NGS is recommended as a supplementary approach for HER2 gene detection. We did not pursue additional fluorescence in-situ hybridization (FISH) testing for this patient, and our decision rested on two main considerations. First, the patient’s IHC result was negative for HER-2 expression; moreover, a study on gallbladder cancer has shown that even cases with IHC 1+ or 2+ did not exhibit HER-2 amplification on subsequent FISH testing (19). In other words, the likelihood of a positive FISH finding was very low given the negative IHC.Second, HER-2 testing for gallbladder cancer is not covered by insurance and must be paid out of pocket. To spare the patient unnecessary expense, we chose not to add the FISH assay — a practical step that aligned with our goal of minimising financial strain while maintaining sound clinical judgment.

T-DM1 is an antibody-drug conjugate (ADC) consisting of trastuzumab (a HER2-targeted antibody) and emtansine (a microtubule inhibitor). The main mechanism of action of the ADC class of drugs is the targeted and efficient delivery of small-molecule cytotoxic drugs into target tumor cells using monoclonal antibodies as carriers. An *in vitro* assay showed that T-DM1 could significantly inhibit biliary tumor cells with high HER2 expression, and the anti-proliferative activity of T-DM1 was positively correlated with HER2 expression (20). This suggests a potential clinical application of T-DM1 in HER2-positive biliary tumors. It is worth noting that accumulating evidence indicates that ERBB2-activating point mutations can confer sensitivity to HER2-targeted ADCs even in the absence of HER2 overexpression or gene amplification (21). The ERBB2 S310F activating mutation disrupts the self-inhibitory conformation of the HER2 extracellular domain (22). This disruption drives constitutive receptor dimerization and subsequently activates downstream signaling pathways (22). Importantly, this mutation represents the most common HER2-driven alteration in gallbladder cancer (23). Tumors carrying this mutation remain sensitive to trastuzumab and HER2-targeted ADCs such as T-DM1 (24). Consequently, for gallbladder cancer patients who do not exhibit HER2 protein overexpression or gene amplification but harbor the ERBB2 S310F activating mutation, the use of T-DM1 as a targeted therapy is supported by a clear molecular rationale (24).

At present, the application of anti-HER2 drugs in GBC is still in the exploratory stage. Clinical data on HER2 gene mutations in GBC are limited, primarily sourced from case reports and gradually evolving randomized controlled trials (**Table 1**). A non-randomized, multicenter phase II clinical trial (MyPathway) evaluated the efficacy and safety of trastuzumab in combination with pertuzumab in HER2-positive BTC. Thirty-nine patients with HER2-positive cholangiocarcinoma were enrolled, and 23% of them achieved partial remission after treatment, with no serious treatment-related adverse events or deaths (25). This study suggests that anti-HER2 drugs may be an option for patients with HER2-positive GBC. Trastuzumab deruxtecan (T-DXd) was evaluated in the DESTINY-PanTumor02 phase 2 basket trial BTC cohort. The overall objective response rate (ORR) was 22% for patients with BTC, rising to 56.3% with a median progression-free survival of 7.4 months among those with IHC 3+ tumours (26). In the phase 2b single-arm HERIZON-BTC-01 study of zanidatamab for previously treated HER2-positive advanced BTC, the confirmed ORR was 41.3% overall and 51.6% in the IHC 3+ subgroup; median overall survival was 15.5 months for the full cohort versus 18.1 months for the IHC 3+ patients (27). Of note, prospective efficacy data for T-DXd and zanidatamab remain limited for BTC tumours harbouring ERBB2-activating mutations without HER2 overexpression, whereas clinical experience with T-DM1 in this rare molecular subset is mainly derived from published case reports (24). .

**Table 1:** Review of reported cases with HER2‐targeted treatment for HER2‐amplified GBC.

Reference	Study type	Diagnosis	HER2/ERBB2 testing details	Treatment regimen	Treatment duration	Outcome	Adverse events
May M et al. (2021) (28)	Case series + literature review	3 patients with metastatic biliary system carcinomas: 2 gallbladder, 1 ampullary; all stage IV	All confirmed HER2-positive: IHC (3+) and/or SISH with copy numbers >12 or 12; no ERBB2 mutations reported	FOLFOX + trastuzumab (→ maintenance 5-FU/trastuzumab); trastuzumab + pertuzumab; CAPOX + trastuzumab (later T-DM1 or other agents on progression)	Ranged from ~7 months (ampullary) to >29 months (one GBC); another GBC continued >20 months	All 3 achieved sustained radiographic response; GBC patient had PR with dual-target therapy (PFS >12 months).	No grade ≥3 AEs reported; main AEs: mild diarrhoea, fatigue.
Lavingia V et al. (2022) (29)	Case report	Metastatic GBC (53-year-old male)	IHC: HER2 (3+) positive; repeat biopsy NGS: ERBB2 amplification (CNV 8)	1st-line: gemcitabine + cisplatin + trastuzumab 2nd-line: mFOLFIRINOX 3rd-line: T-DM1 monotherapy	T-DM1: ~6 months	T-DM1 provided ~6-month survival benefit after progression on prior therapies.	T-DM1 well-tolerated; no significant AEs reported.
Wang L et al. (2021) (30)	Case report	Metastatic GBC with resistance to trastuzumab + chemotherapy	Methods: IHC, FISH, NGS. Results: IHC 3+, FISH+ (HER2/CEP17 ratio >2.0); NGS ERBB2 amplification (fold change 8.7).	Trastuzumab + chemotherapy (progression) addition of camrelizumab (PD-1 inhibitor)	Combination immunotherapy: >6 months	Complete regression of all metastases (lung, liver, brain) after 5 cycles; CA199 normalised; KPS improved from 80 to 90; sustained complete response at last follow-up	Mild rash and fatigue; no grade ≥3 immune-related AEs.
Mondaca S et al. (2019) (24)	Retrospective cohort + case report	517 biliary tract cancer (BTC) patients (313 ICC, 93 EHC, 111 GBC)	Methods: NGS Results: ERBB2 alterations in 5.4% (28/517): 2.7% amp, 2.3% mut, 0.4% both. GBC highest rate (12.6%); median amp copy number 6.4; most common mutation: S310 codon (ECD).	T-DM1 (1 patient with ERBB2-amplified extrahepatic cholangiocarcinoma)	T-DM1 given until progression	PR, PFS was 8.6 months	Not reported in detail.
Javle M et al. (2015) (31)	Retrospective analysis	BTC patients with HER2 overexpression/amplification (mixed GBC, cholangiocarcinoma)	IHC (3+), FISH (ratio ≥2.0), or NGS (amplification/mutation). GBC: 8/9 had amplification/overexpression, 1 had V777L mutation. CCA: 2 had mutations (S310F, V777L), 3 had amplification.	Trastuzumab alone or combined with chemotherapy	GBC: 8–168 weeks (median 40 weeks); CCA: 6–19 weeks	GBC: CR (1), PR (4), SD (3), MR (1); CCA: all 5 had PD; median OS: GBC 62–178 weeks, CCA 12–29 weeks	HER2-directed therapies were well-tolerated; no cardiac events reported despite prolonged therapy
Sorscher S (2013) (32)	Case report	Metastatic gallbladder adenocarcinoma	IHC: HER2 strongly positive in majority of tumor cells, equivalent to 3+ by breast/gastric criteria	Trastuzumab monotherapy	>4 months	Radiographic response: largest liver lesion decreased from 8.5×5 cm to 1.3×0.8 cm	Well-tolerated; no significant infusion-related reactions.
Inagaki C et al. (2019) (33)	Case report	GBC,primary pT2N0M0, later metachronous liver metastasis	NGS (Oncomine): ERBB2 S310F (c.929C>T) activating mutation – VAF 18% in primary, 26% in liver metastasis; IHC: HER2 0 (negative)	Prior: gemcitabine + cisplatin → TS-1 (PD). Then: lapatinib (1250 mg/day) + capecitabine (2000 mg/m² days 1–14, q21d)	4 cycles; discontinued due to mucositis	Clinical improvement (edema resolved) within 1 week; after 2 cycles: hepatic lesions and tumor emboli decreased; after 4 cycles	Grade 3 mucositis (led to discontinuation); resolved within 2 weeks off treatment
Ye M et al. (2019) (34)	Case report	Recurrent metachronous metastatic GBC (brain and lung metastases)	NGS (416-gene panel) on primary tumour, brain metastasis, and ctDNA: ERBB2 amplification – primary 8.4×, brain 14.6×, ctDNA 2.48×;	Trastuzumab + lapatinib (dual blockade)	>6 months	Brain and lung metastases achieved PR.	Grade 3 fatigue, skin rash, and increased bilirubin; no grade 4/5 events
Nam AR et al. (2016) (35)	Prospective cohort study with preclinical experiments	Advanced BTC patients and cell lines	IHC and FISH; HER2-positive defined as IHC 3+ or FISH ratio >2.0. Prevalence: 13.0% (6/46) in cohort; all were gallbladder cancer	Trastuzumab-based chemotherapy: combined with gemcitabine/cisplatin, paclitaxel, or capecitabine/cisplatin; one patient also received a novel anti-HER2 antibody after progression	PFS on trastuzumab-based regimens: 12–25 weeks; one patient had PFS of 34 weeks on novel antibody	Best responses on trastuzumab-based therapy: SD (2 patients), PR (1 patient); one additional PR on novel antibody. Overall, HER2-positive patients tended to have longer OS (20.6 vs. 13.8 months, not significant)	Not specified; generally well-tolerated

ADC, antibody-drug conjugate; AE, adverse event; BTC, biliary tract cancer (including gallbladder carcinoma, cholangiocarcinoma, and ampullary carcinoma); CAPOX, capecitabine plus oxaliplatin; CCA, cholangiocarcinoma; CNV, copy number variation; CR, complete response; ctDNA, circulating tumour DNA; EHC, extrahepatic cholangiocarcinoma; FISH, fluorescence *in-situ* hybridisation; FOLFOX, 5-fluorouracil, leucovorin, and oxaliplatin; FOLFIRINOX, 5-fluorouracil, leucovorin, irinotecan, and oxaliplatin; GBC, gallbladder cancer; GP, gemcitabine and cisplatin; GTX, gemcitabine, docetaxel, and capecitabine (or other platinum-based triplet); IHC, immunohistochemistry; ICC, intrahepatic cholangiocarcinoma; KPS, Karnofsky Performance Status; mFOLFIRINOX, modified FOLFIRINOX (with reduced doses of irinotecan and/or oxaliplatin); MR, mixed response; NGS, next-generation sequencing; OS, overall survival; PD, progressive disease; PD-1, programmed cell death protein-1; PFS, progression-free survival; PR, partial response; RECIST, Response Evaluation Criteria in Solid Tumours; SD, stable disease; SISH, silver-enhanced *in-situ* hybridisation; T-DM1, trastuzumab emtansine (ado-trastuzumab emtansine); TMB, tumour mutational burden; VAF, variant allele frequency; XP, capecitabine and cisplatin.

However, when evaluating the potential of T-DM1 in GBC, an important consideration is the racial differences in efficacy and toxicity. Although data on racial differences in the efficacy of T-DM1 for GBC are currently very limited, studies on racial differences in the breast cancer field have shown no significant differences in efficacy trends and toxicity between Asian and non-Asian patients (36). However, there are currently no clinical studies on T-DM1 for the treatment of GBC. In Asian populations, the incidence of GBC is relatively high, and there may be racial differences in the HER2 positivity rate (37). This case involves an Asian patient, but single-case data do not reveal racial patterns. Therefore, further studies specifically evaluating T-DM1 for HER2-positive GBC in different populations, including Asian cohorts, are crucial for fully understanding its applicability across different racial groups.

On reviewing the previous treatment history of this patient, he was a middle-aged man diagnosed with stage IVB GBC (T4N0M1) with no indication for surgical treatment. The TOPAZ-1 and KEYNOTE-966 clinical trials have successively demonstrated that immune checkpoint inhibitors combined with chemotherapy can significantly improve patients’ progression-free survival and overall survival (38, 39). However, this patient showed stable disease after receiving two cycles of gemcitabine, oxaliplatin, lenvatinib, and sintilimab, failing to meet the expected efficacy. Given the suboptimal response to chemotherapy combined with immune-checkpoint inhibitors, the patient underwent supplementary testing for immune-related markers, including a CPS of 1%, MSS, and TMB of 4.87 mutations/Mb. However, current clinical data indicate that immune-checkpoint inhibitors have demonstrated survival benefits in biliary tract malignancies, irrespective of whether the CPS score is <1 or ≥1 (38, 39). NGS results showed the patient was HER2-positive, and considering the patient’s young age and strong treatment desire, there was a potential indication for HER2-targeted therapy. Current clinical evidence indicates that systemic therapy for biliary tract malignancies remains combination therapy primarily based on chemotherapy. Based on the above, the MDT team formulated a treatment regimen of trastuzumab emtansine combined with sintilimab.

## Conclusions

GBC is a malignant tumor that arises from the adenoepithelium of the gallbladder. Early-stage GBC can be cured by radical surgical treatment, while intermediate- and late-stage GBC is usually treated with a combination of therapies, with chemotherapy still being the mainstay. However, with chemotherapy, most patients are not able to achieve surgical conditions. In our case, the patient was treated with T-DM1 for advanced metastatic HER2-positive GBC, achieving the requirement for surgical resection and a disease-free survival of up to 13 months after surgery. This case highlights the possibility of exploring T-DM1 for the treatment of advanced metastatic HER2-positive GBC.

## Data Availability

The original contributions presented in the study are included in the article/supplementary material. Further inquiries can be directed to the corresponding authors.
